# Defining displacement thresholds for surgical intervention for distal radius fractures – A Delphi study

**DOI:** 10.1371/journal.pone.0210462

**Published:** 2019-01-08

**Authors:** Nick Johnson, Paul Leighton, Distal Radius Fracture Delphi Study Group, Charles Pailthorpe, Joseph Dias

**Affiliations:** 1 Academic Team of Musculoskeletal Surgery, University Hospitals of Leicester, Leicester, United Kingdom; 2 University of Nottingham, Queen's Medical Centre, Nottingham, United Kingdom; 3 Royal Berkshire Hospital, Reading, United Kingdom; Augusta University, UNITED STATES

## Abstract

Distal radius fractures are very common yet controversy exists regarding which require treatment and is reflected by significant variation in surgical intervention rate. Evidence regarding which fractures would benefit from intervention is varied and largely poor quality. This study had three aims; identify which radiographic parameters are clinically important; quantify the threshold of displacement at which intervention should occur and investigate which patient factors influence the decision to intervene. A modified three round Delphi study was carried out and responses were qualitatively analysed. The Delphi panel was composed of three groups of national and international expert surgeons: hand and wrist surgeons, trauma surgeons, and international researchers. 46 participants initially agreed to take part. 43 completed the first round and all then completed three rounds. Participants were asked questions based around case vignettes in patients of three ages (38, 58, 75 years). For all age groups ulnar variance was ranked as the most important extra-articular parameter, step was ranked as the most important intra-articular parameter. Agreed thresholds were the same for all parameters for patients aged 38 and 58. Surgeons would intervene with +2 mm ulnar variance, 10 degrees dorsal tilt, 2mm step and 3mm gap. In patients aged 75 the agreed thresholds were 20 degrees dorsal tilt, 3mm step and 4mm gap, consensus was not achieved for ulnar variance.

Mental capacity, pre-injury functional level and medical co-morbidities were ranked as the most important factors influencing the decision to intervene. Qualitative analysis suggested that pre-injury function was the main theme within these factors. Our findings provide useful advice about which parameters should be measured and radiographic thresholds for intervention. These thresholds may then be modified depending on important patient factors. This information can help guide clinicians with management decisions and reduce variation.

## Introduction

Distal radius fractures (DRF) are the most frequently treated fracture worldwide and a huge burden on healthcare resources. Around 70,000 individuals are affected each year in the United Kingdom (UK).[1] Incidence continues to rise with an ageing population.[2] Despite the frequency in which these injuries are encountered optimum treatment remains controversial. Many fractures may require only simple, safe, low cost interventions such as cast immobilisation. Displaced or intra-articular injuries are often treated surgically with manipulation and Kirschner wire (K-wire) fixation, or with open reduction and internal fixation using locking plates. Plate fixation is significantly more expensive [3]and has become an increasingly common management strategy[4]. Randomised trials have not shown a benefit of locking plates compared to K-wire fixation for fractures that can be reduced closed. [5, 6] Several meta-analyses combining these results have generated conflicting findings. [7–9]

Significant variation in the rate of surgical intervention for DRF exists. Studies from the United States have shown an almost ten fold variation in fixation rate depending on hospital region with similar results reported from the Netherlands. [10, 11] This variation may be warranted or unwarranted. Warranted variation may demonstrate high quality clinical care. Rates of surgical intervention for DRF can justifiably vary due to individual needs of specific patient populations.[12] Areas with large populations of older patients may have increased surgical fixation rates due to a high incidence of displaced fragility fractures. Unwarranted variation suggests low quality healthcare with activities of low value and waste of resources.[13, 14] Over use of expensive and invasive surgical interventions such as plate fixation potentially exposes patients to risk from non-essential treatment, wastes resources and diverts funding from other patients and more appropriate treatments.

When deciding whether to intervene for a patient with a displaced distal radius fracture a clinician will usually measure displacement parameters on plain radiographs. Many different measurable parameters have been described and reported to be of use for clinical decision making and predicting outcome after distal radius fracture.[15] The evidence base for this is inconsistent and varied. Several studies have shown a persistent intra-articular step after bony union leads to progressive radiocarpal joint arthritis and poorer outcome. However others have shown no difference. Kreder et al demonstrated no difference in functional outcome at 1 year in patients with intra-articular incongruity who had been treated with plate fixation.[16] Kopylov and Forward reported minimal impact on functional outcome in patients reviewed 30 and 38 years after intra-articular fractures respectively.[17, 18] Similar disagreement regarding radiographic findings and outcome can be found in the literature for the other radiographic parameters. Recently Plant et al used data from a prospective randomised trial to investigate the correlation between palmar tilt and ulnar variance with functional outcome measures.[19] No correlation was found and they questioned whether restoration of 'normal' radiographic parameters was necessary to achieve a satisfactory functional outcome for the patient.

We conducted a systematic review following Scottish Intercollegiate Guidelines Network (SIGN) guidelines looking at literature which investigates the relationship between measured radiological parameters and functional outcome[20]. A large number of studies were identified most of which were retrospective. There was significant heterogeneity and assessment of quality revealed the majority to be low quality. The blue book committee, which is a multi-disciplinary group commissioned by the British Orthopaedic Association (BOA) and the British Society for Surgery of the Hand (BSSH), to provide national guidance for clinicians treating distal radius fractures considered the output of the systematic review and concluded that currently there is insufficient evidence to demonstrate an association between any measured radiological parameters and patient rated outcome. The review identified that the most commonly measured parameters were radial height, radial inclination, volar tilt, ulnar variance and intra articular step and gap but could not help define which of these were important or what the acceptable displacement threshold should be, these aspects were considered essential by the blue book committee.

We therefore carried out a Delphi study with a panel of national and international experts who have experience in treating acute distal radius fractures and their longer term sequalea and/or have published clinical research investigating outcome after DRF. The Delphi technique is a structured method of obtaining expert opinion and has been widely used in health research to define diagnostic criteria, treatment components, patient pathways, research priorities and research outcome measures across a broad range of clinical areas. Participants provide answers to research questions anonymously in a series of rounds with aggregated feedback provided after each round. They may then adjust their answers in subsequent rounds following review of the group feedback. Consensus on the research questions is sought according to pre-defined criteria. Where definitive evidence is lacking, the Delphi approach challenges an expert panel to reach agreement on a research question. It draws together knowledge and experience from a number of experts but prevents one voice from controlling the agenda.

The study had three aims; to identify which radiographic parameters the participants felt to be clinically important; quantify the threshold of displacement at which they would intervene surgically for the commonly measured displacement parameters and investigate which patient factors would influence their decision to intervene. This information can then help guide clinicians with their management decisions for this very common injury and reduce variation in treatment.

## Method

Full ethical approval was obtained from the University of Leicester (Reference: 9559-nj94-healthsciences). The protocol was registered with ClinicalTrials.gov (Identifier: NCT03126474).

The protocol and first round questions were presented to the BSSH blue book for distal radius fracture management committee and feedback obtained.

### Delphi panel recruitment

The panel was composed of 3 groups of expert surgeons. Many panel members fitted the criteria to belong in more than one group.

1 –Hand and wrist specialists–these surgeons would have considerable experience dealing with acute injuries and longer term problems after DRF. Practicing hand and wrist consultant surgeons were identified by sampling from BSSH members geographically by UK regions.
2 –Trauma surgeons- those who deal acutely with patients with DRF and operate on them regularly. An email invitation was sent via the Orthopaedic Trauma Society (OTS) asking for volunteers to take part who operated on at least 20 acute distal radius fractures per year.
3 –International researchers–practicing surgeons who are also researchers so have a comprehensive understanding of the nature of the injury and likely outcome. A literature review of major orthopaedic and hand journals was carried out to identify researchers who have published studies investigating functional outcome in patients with DRF in the last 2 years.

Experts were invited to take part by email. No incentives were offered to participants.

### Questions

Questions were based around 6 short case vignettes regarding a displaced extra-articular fracture in a 38, 58 and 75 year old patient, followed by a displaced intra-articular fracture in the same age groups (S1 and S2 Appendices). Those age groups were selected as we felt they would stimulate greater thought about decision making than if we had used more extremes of age. No further clinical details or any images were provided to prevent participants allowing other factors to influence their decision. Intervention was defined as any type of reduction and stabilisation including manipulation and cast application.

Participants were asked to consider the functional outcome for each patient at 3 months following injury. This time period was chosen as the fracture should have united and the patient would have been free of splintage and mobilising the wrist for approximately 6 weeks.

A summary of results and comments were produced and sent to each panel member after each round. Quantitative group results for each question (median, mean, quartiles, minimal, and maximal ratings) were provided within the survey for the subsequent round. An individual summary of each participant’s own responses was provided, to illustrate their position compared to the rest of the panel, along with an anonymous abstract of panel members’ comments. In round 3 a selection of systematically reviewed literature was presented to the participants to help aid decision making (S3 Appendix).

### Importance of parameters

Participants were asked to rank the parameters in order of importance on a visual analogue scale of 0 to 10 (0 = extremely unimportant, 10 = extremely important). Parameters were then ranked by median score and the results presented to the panel. Participants were asked if they agreed with the ranking. Consensus was defined as at least 70% agreement between participants.

### Thresholds for intervention

For each case vignette panellists were asked at what measurement of displacement for each parameter they would intervene surgically. Agreement was then sought on the value at which intervention is required for each parameter by presenting the median value (from those scores independently offered in round one) alongside a scale of greater and lesser values. Median values were presented for the continuous measured values (angles and lengths) to reduce potential skew from outliers and avoid problems due to averaging, rounding up or down. We know that in answering such questions clinicians prefer certain digits (e.g. multiples of 5 degrees, and certain mm intervals). Where 70% agreed we accepted this as the threshold at which intervention should take place.

### Patient factors influencing decision making

Ten patient factors which may influence decision making were identified after discussion with the blue book committee. The factors were presented to participants who were asked to rate how important the factor is when deciding to intervene on a visual analogue scale of 0 to 10 (0 = extremely unimportant, 10 = extremely important). Factors were then ranked in order of importance by median score. A factor with a median score of 3 or less was accepted to be not important. A median score with an inter-quartile range of 2 or less was accepted as consensus for that score. Participants were asked if they agreed with the ranking. Consensus of the ranking was defined as at least 70% agreement between participants.

Free text comment boxes were provided throughout and participants were asked if they felt any other parameters or factors should be included. Further free text questions to investigate participant’s decision making were asked in round three.

A simple thematic analysis, informed by a framework approach was applied to the qualitative, free text answers and comments.[21] Data was mapped to a framework consisting of the patient factors already identified for scoring (as described above). New factors were added to the framework, and existing factors refined, where comments offered new and more nuanced perspectives. For example, ‘age’ was refined to capture comments relating to ‘age–definition’ and ‘age–not pertinent’; the ‘function’ code developed to incorporate ‘function–definition’, ‘activity levels’, ‘occupation’, and so on. Review of the populated framework offered additional insight to interpret the Delphi scoring.

The Delphi rounds were created using SurveyMonkey software. For each round the survey link was emailed to participants and was online for 2 weeks. Reminder emails were sent 7 days after each round began to those who had not completed the survey. Only those who completed each round were invited to participate in the next round.

## Results

### Participant responses

**Fig 1:**
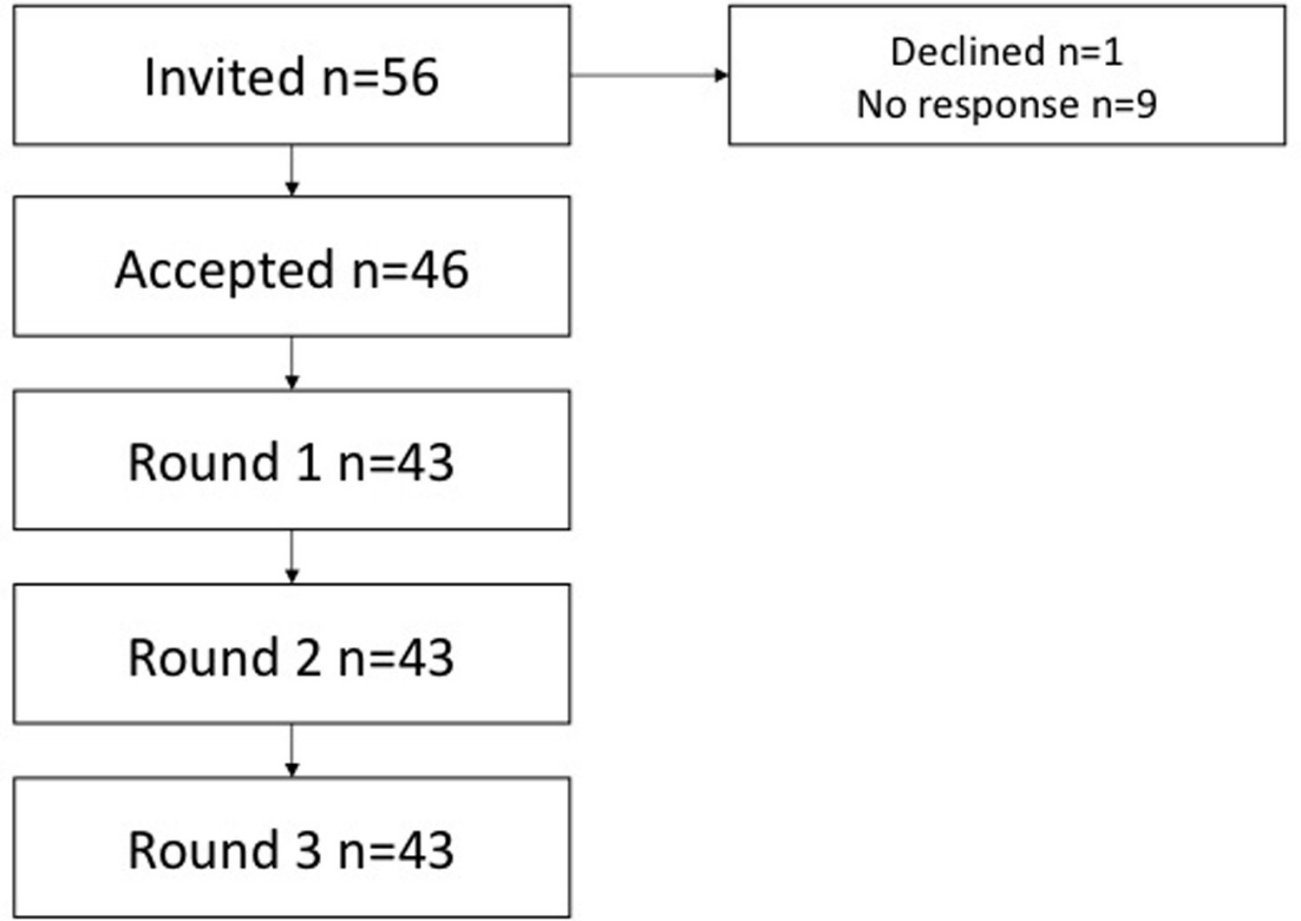
Flow chart demonstrating numbers of invited participants and those who completed all rounds of the Delphi process.

100% (43) of participants who took part in round 1 completed all rounds of the Delphi study. Fig 1 illustrates the response rates throughout the study (Fig 1). 17 participants were hand and wrist surgeons, 14 were trauma surgeons and 12 were researchers. All participants currently practice and treat patients with DRF. The hand and wrist surgeons and trauma surgeons all practice in the UK. Four researchers were from the UK, three from Europe, two from the United States and one each from Canada, China and Australia. Responses from rounds one and two can be found within the surveys from the subsequent rounds (S2 and S3 Appendices).

### Importance of parameters

**Fig 2:**
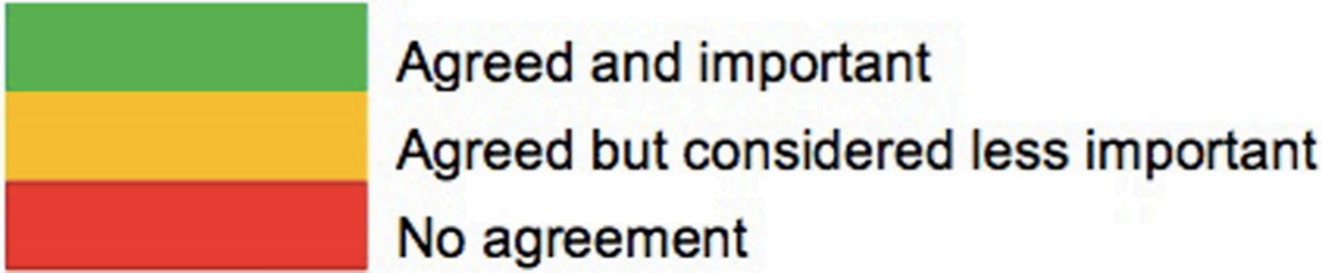
The following traffic light system is used to illustrate agreement and importance.

Ulnar variance was consistently rated as the most important extra-articular parameter with dorsal tilt rated as the second most important for all age groups (Fig 2 and Table 1). Step was rated as the most important intra-articular parameter for all age groups. The panel agreed with these rankings orders for all parameters.

**Table 1:** Ranking by importance of extra and intra-articular radiographic parameters.

	Parameter	
Ranking	Extra-articular	Intra-articular
1	Ulnar variance	Step
2	Dorsal tilt	Gap
3	Radial inclination	
4	Radial height	

### Ranking of importance of radiographic parameters

#### Thresholds for intervention

Tables 2 and 3. Radiographic threshold at which surgeon’s would intervene. Agreement denotes the percentage of the expert panel who would intervene at this measurement.

**Table 2:** 

	Parameter			
Age	Ulnar variance (mm)	Dorsal tilt (degrees)	Radial inclination (degrees)	Radial height (mm)
38	3	10	10	5
agreement	84%	79%	90%	85%
58	3	10	10	5
agreement	74%	87%	82%	90%
75	4 / >5	20	10	5
agreement	50% / 42%	87%	91%	88%

**Table 3:** 

	Parameter	
Age	Step (mm)	Gap (mm)
38	2	3
	81%	84%
58	2	3
	76%	87%
75	3	4
	76%	79%

Consensus was obtained for all thresholds for intervention except ulnar variance in a 75 year old patient. For this case half of the panel would intervene at 4mm of positive ulnar variance whereas 42% would accept over 5mm of displacement. Agreed thresholds were the same for all parameters for patients aged 38 and 58. There was no difference seen in thresholds between the 3 groups of participants.

### Patient factors

Seven parameters were identified as important by the panel (Table 4). Alcohol intake, smoking and gender were judged to be not important. Consensus was gained on the following rank order:

**Table 4:** Rank order by importance of patient factors which influence a surgeon’s decision to intervene.

1	Mental capacity
2	Function
3	Medical co-morbidities
4	Age
5	Compliance with rehabilitation
6	Occupation
7	Fragility

Stability was seen between rounds indicating that panel members did not significantly change their choices throughout the rounds. There was no difference seen in preferences between the 3 groups of participants.

### Qualitative review

A number of comments in round 2 stressed that thresholds for intervention should not be absolute, but rather be considered as a framework within which a clinician might make tailored decisions for each patient.

“an 'average' number belies the fact that there is a great deal of disagreement about what is really important for patients. I'm against the idea of setting arbitrary radiographic thresholds based on the average view”

More explicit exploration of this notion in round 3 demonstrated a more nuanced understanding of each of those factors ranked above. Restoring ‘function’ was presented as a more important over-arching criteria for guiding treatment (Table 5):

“I place importance on the functional level and use the discussion of activities … to determine the ability to tolerate deformity and need for intervention”

Comments about other factors frequently reinforced the priority offered to a more general assessment of function:

Age: “I think pre-injury function is a more reliable parameter than absolute age.”

Mental capacity: “important in terms of consent and rehab, but also often affects the daily function and therefore requirements of the healed wrist.”

Fragility/co-morbidities: “I don’t think it’s really about co-morbidity per se—more about frailty. For me, this is to do with patient expectation of functional recovery”.

**Table 5:** Themes and subthemes identified in the free text questions, presented alphabetically by theme.

Theme:	Sub themes:	Lower order themes:
Age [67 data points]	- Defining age - Age a pertinent factor in decision making - Age NOT a pertinent factor in decision making	
Compliance [57 data points]	- Compliance as important - Compliance as a problem - Practical barriers to compliance	
Comorbidities [19 data points]	- Comorbidities a pertinent factor in decision making. - Comorbidities NOT a pertinent factor in decision making	
Function [147 data points]	- Function important in decision making - Defining function - Occupation - Activity type and level pertinent to decision making	- Independence (as definition)
Mental Capacity [122 data points]	- Mental capacity as a pertinent factor in decision making - Mental capacity NOT a pertinent factor in decision making	
Subjective judgement [9 data points]	- Clinician’s judgement is subjective - Patient preference is important	

## Discussion

Through this Delphi process our panel of experts agreed that ulnar variance and dorsal tilt are the most important extra-articular parameters and step is the most important intra-articular parameter.

Agreed thresholds were the same for all parameters for patients aged 38 and 58. Surgeons would intervene in patients aged 38 and 58 with +2 mm ulnar variance, 10 degrees dorsal tilt, 2mm step and 3mm gap. In patients aged 75 the agreed thresholds were 20 degrees dorsal tilt, 3mm step and 4mm gap, consensus was not achieved for ulnar variance. Seven patient factors were thought to be important regarding whether to intervene surgically and rank order of importance was agreed for these.

Mental capacity was ranked as the most important factor influencing the decision to intervene. In round 3 participants were asked why they thought mental capacity was ranked first. It was clear from the qualitative analysis that the most important aspect affecting decision making was actually function. Overall function was identified as the key factor guiding treatment with restoration of functional level the key aim. Assessment of pre-injury functional level is therefore vital. Function itself is multilayered and complex and difficult to define and evaluate. Our panel suggested they would assess function by discussion with the patient along with a carer or family members, this is supplemented by assessment of some basic facts such as handedness, activities of daily living, as well as a subjective assessment of the discussion and patient history. Independence was also judged to be important.

Mental capacity itself was seen as a pertinent factor in 3 distinct ways: its effect on functional activity, post operative compliance and the influence on a patient’s decision making. Age was used descriptively as a factor in treatment decisions, although individual circumstances and functional level were presented as more important factors. Functional level was again recognised as a relevant factor with regards medical co-morbidities. If a procedure was not to be undertaken in a patient with multiple comorbidities this was more likely to be due to a low level of function rather than the actual comorbidities themselves.

The other theme that developed around medical co-morbidities was fitness for anaesthesia and potentially changing anaesthetic technique and/or intervention method. Such as performing a manipulation under haematoma block rather than surgical fixation under general or regional anaesthesia due to the patient having multiple co-morbidities. Compliance was felt to be important in two ways: adherence to post operative rehabilitation and the possibility of a non compliant patient compromising an intervention by interfering with a wound or removing a cast. Fragility was not considered to be an influence on whether to intervene or not but rather on the method of intervention. Many participants suggested they would choose to use a locking plate in osteoporotic bone to increase the chance of obtaining stable fixation.

**Fig 3:**
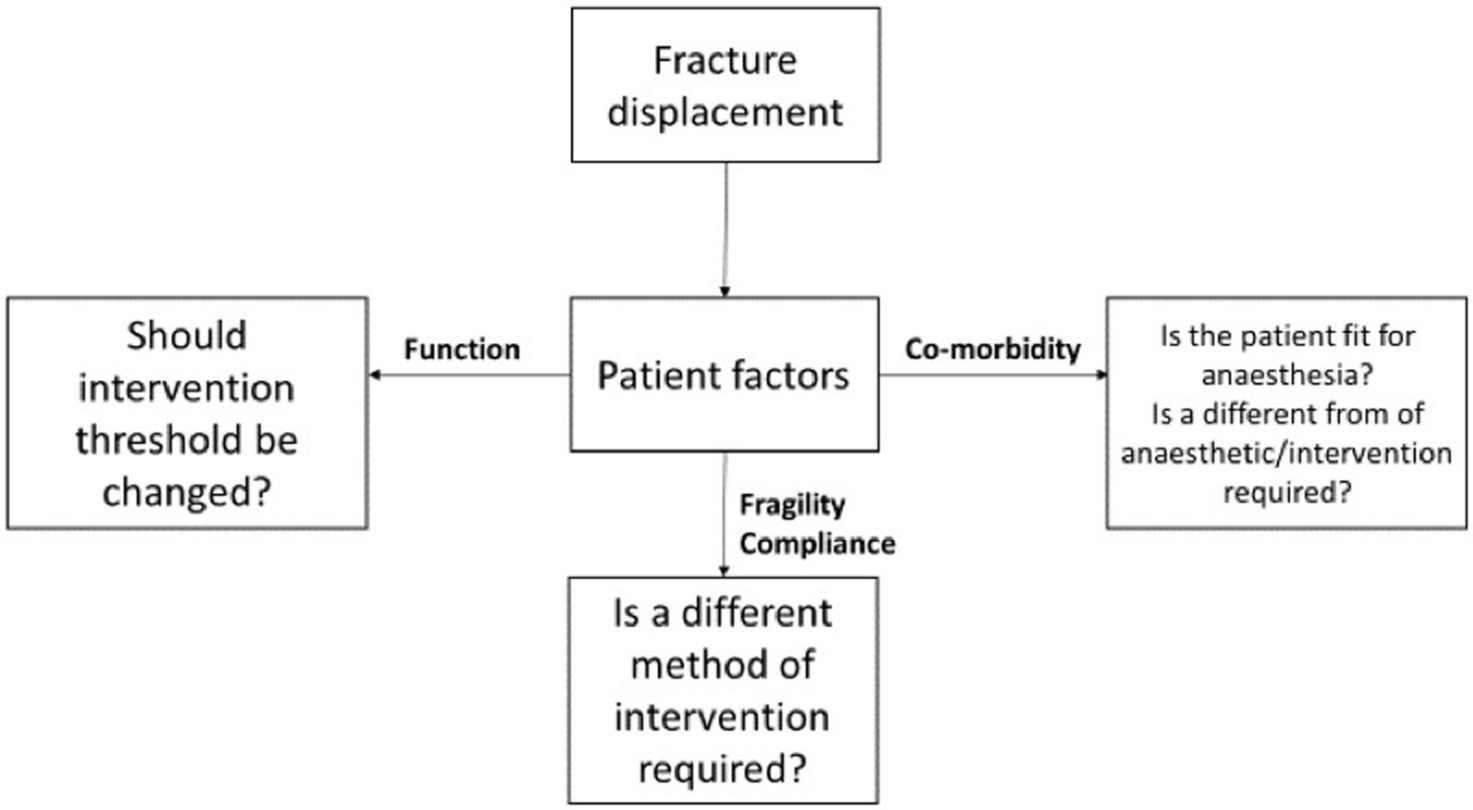
Flow chart demonstrating factors influencing surgeon’s decision making when planning intervention for a patient with a distal radius fracture.

The decision making steps we have identified are summarised in Fig 3. Following an initial review of the radiographs a surgeon decides whether intervention may be required based on radiographic thresholds. After reviewing the patient and discussing treatment options the treatment thresholds may be increased or decreased based on the patients level of function. Function is a complex assessment based on many factors including age, occupation, mental capacity and medical co-morbidities. If intervention is deemed necessary the next step is to decide whether the patient is fit for anaesthesia and should the type of intervention be changed. Maintenance of the reduction following intervention is then considered which is influenced by compliance and fragility.

Carpal malalignment/displacement was identified as an additional important radiographic parameter by 74% of participants. Carpal malalignment is defined as the displacement of the longitudinal axis of the capitate either dorsal or volar to the longitudinal axis of the radius.[22] It is related to loss of volar tilt after DRF. Change in the orientation of the distal articular surface of the radius adversely influences the intercarpal relationship with loss of volar tilt causing the lunate to dorsiflex and the capitate go into a compensatory flexion pattern.[23] Several studies have shown it is associated with poorer functional outcome.[24, 25] Correction of dorsal angulation leads to reduction of the carpal malalignment. Using our identified thresholds dorsal tilt of 10 degrees in 38 and 58 year olds and 20 degrees in older patients would be corrected. This would therefore lead to reduction of associated carpal malalignment for those patients. We asked panelists what they would do in a situation in which a patient with an acute DRF with less than 10 degrees of dorsal tilt had carpal malalignment. Panelists felt this would be an unlikely scenario but they would be likely to intervene by reducing the dorsal tilt in this situation to also correct the carpal malalignment.

Currently significant variation exists in the rate of surgical intervention for DRF. Despite DRF being the most commonly treated fracture in the world there is limited evidence about which patients benefit from surgery and the value of more expensive fixation methods. This Delphi study has utilized 614 years of national and international expert knowledge to provide guidance for clinicians treating DRF. We strictly followed a methodology based on recent evidence for producing and reporting high quality Delphi studies.[26] Up to date recommendations are provided utilising a more robust method than previous guidelines from national expert groups[27, 28]. Our findings provide useful advice about which parameters should be measured and thresholds for intervention. These thresholds may then be modified depending on the relevant and important individual patient factors. They can be used for audit and we have identified further research questions. This information can help guide clinicians with their management decisions, reduce variation and ensure patients are receiving effective, cost efficient, safe treatment.

## Supporting information

S1 Appendix Round 1 survey. (PDF)

S2 Appendix Round 2 survey. (PDF)

S3 Appendix Round 3 survey. (PDF)
