# Supplementary material for: Defining displacement thresholds for surgical intervention for distal radius fractures – A Delphi study
Source: PLoS One. 2019 Jan 8;14(1):e0210462. doi: 10.1371/journal.pone.0210462 (PMC6324814; doi:10.1371/journal.pone.0210462)
Supplement: S1 Appendix — (PDF) [file pone.0210462.s001.pdf]

Defining displacement thresholds for surgical intervention for distal radius fractures - a Delphi study

ATOMS
Academic Team of Musculoskeletal Surgery

Introduction

Thank you for agreeing to take part in this Delphi study.

The aim of the study is:
To investigate surgeon's opinions and obtain consensus regarding how much displacement shouldbe accepted in distal radius fractures before intervention is required.

We will aim to quantify how much displacement can be accepted before intervention is required for different patient groups.

This will provide guidance and reduce variation between treating surgeons.

There will be **3 rounds** of questionnaires based around **6 simple case vignettes**. Each round will take place approximately **4 weeks** following the previous round.

Feedback will be provided throughout. We estimate each questionnaire will take **less than 20 minutes** to complete and are likely to get shorter with each subsequent round.

By intervention, we mean: any form of manipulation or surgical fixation.

For each case, please consider the functional outcome at **3 months post injury** in a patient with no *complications*, who had *no wrist problems* and *normal wrist anatomy* before the injury.

Any data you give will be protected and secured confidentially by the research team.

We are extremely grateful for your time and support.

Mr Nick Johnson
Chief Investigator
Honorary Academic Fellow Trauma & Orthopaedics

Professor Joseph Dias
Professor of Hand & Orthopaedic Surgery
University Hospitals of Leicester

1. Please enter your name.

ATOMS
Academic Team of Musculoskeletal Surgery

Defining displacement thresholds for surgical intervention for distal radius fractures - a Delphi study

Extra-Articular Fractures of the Distal Radius

This page asks about the factors affecting **Extra-Articular** Fractures. There are 3 cases of patients of varied ages.

The radiological parameters used in these 3 cases are described below.

When providing a measurement at which you would intervene give the **absolute value** for that radiological measurement.

Please use + or – to indicate positive or negative ulnar variance and negative values to indicate dorsal tilt.

Please assume all patients in the cases had normal wrist anatomy *before injury* with parameters of the following values:

Radial height: 11 mm
Ulnar variance: 0 mm
Radial inclination: 22 degrees
Dorsal tilt: 11 degrees
Intra-articular gap/step: nil

Radial height

On a PA view this is the vertical distance between:

- the tip of the radial styloid
- a line tangential to the ulnar corner of the lunate fossa

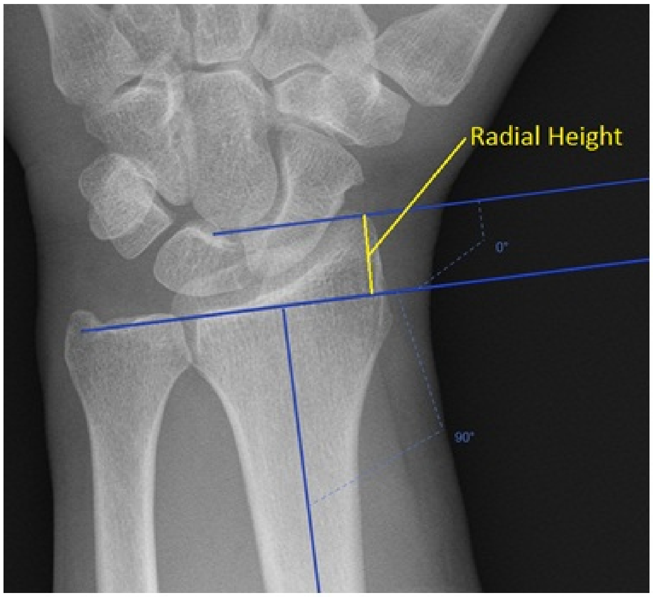

Ulnar variance

On a PA view this is the vertical distance between:

- a line tangential to the articular surface of the ulna and perpendicular to the long axis of the radius
- a line tangential to the ulnar corner of the lunate fossa of the radius and perpendicular to the long axis of the radius

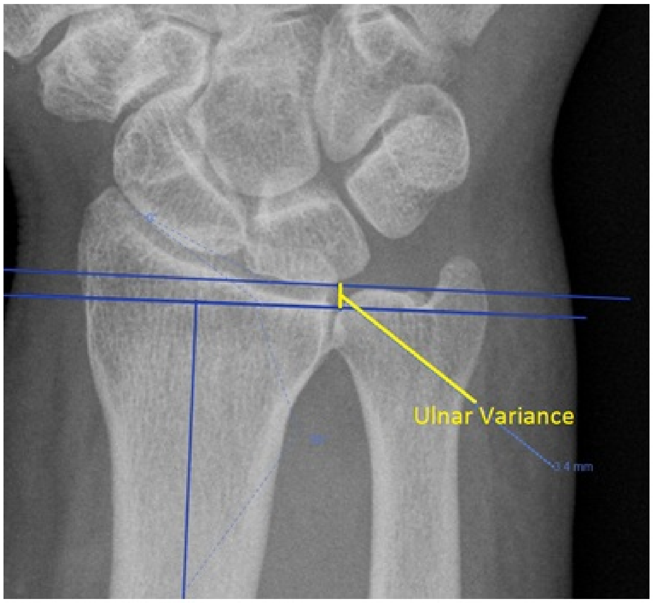

Radial Inclination

On a PA view this is the angle between:

- a line drawn from the tip of the radial styloid to the medial edge of the articular corner of the radius
- a line perpendicular to the long axis of the radius

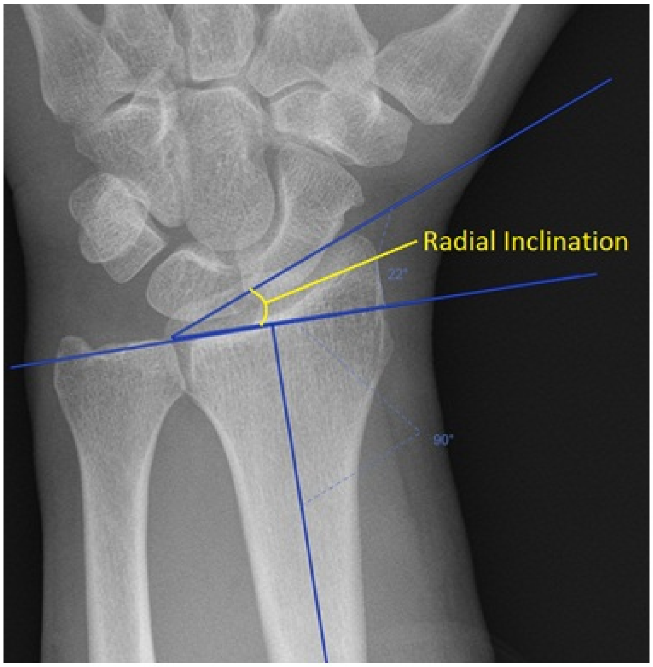

Dorsal tilt

On a lateral view this is the angle between:

- a line drawn between the most distal points of the dorsal and volar lips of the distal radius
- a line perpendicular to the long axis of the radius

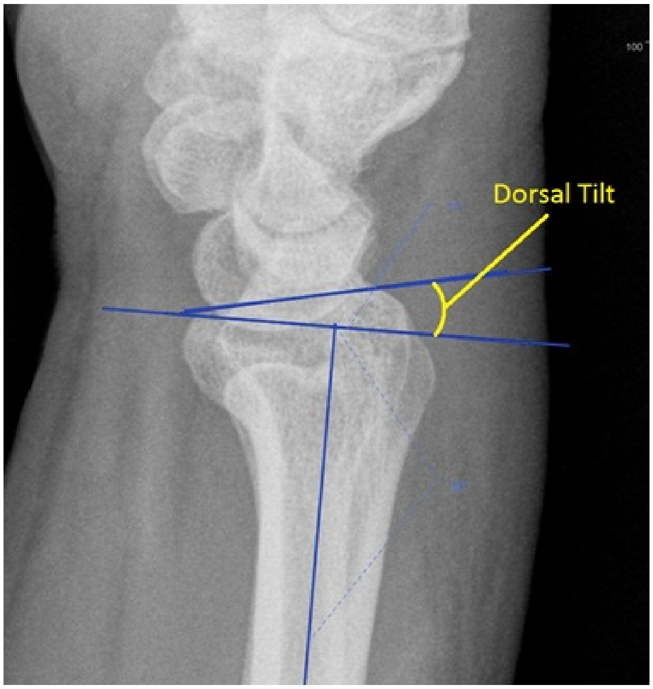

Case 1: A **38 year old** patient sustains a displaced **extra-articular** fracture of the distal radius.

* 2. How important is restoring **radial height** to the position prior to injury to prevent an adverse effect on functional outcome for this patient?

Please rate on the scale (0=extremely unimportant, 10=extremely important)
Click on the line at the appropriate position, or enter a numerical rating in the textbox.

0	Importance of Radial Height	10

* 3. How important is restoring **ulnar variance** to the position prior to injury to prevent an adverse effect on functional outcome for this patient?

Please rate on the scale (0=extremely unimportant, 10=extremely important)
Click on the line at the appropriate position, or enter a numerical rating in the textbox.

0	Importance of Ulnar Variance	10

* 4. How important is restoring **radial inclination** to the position prior to injury to prevent an adverse effect on functional outcome for this patient?

Please rate on the scale (0=extremely unimportant, 10=extremely important)
Click on the line at the appropriate position, or enter a numerical rating in the textbox.

0	Importance of Radial Inclination	10

* 5. How important is restoring **dorsal tilt** to the position prior to injury to prevent an adverse effect on functional outcome for this patient?

Please rate on the scale (0=extremely unimportant, 10=extremely important)
Click on the line at the appropriate position, or enter a numerical rating in the textbox.

0	Importance of Dorsal Tilt	10

6. At what measurement of displacement of **radial height** would you intervene surgically?

- [ ] I **would not** intervene (irrespective of the amount of displacement)
- [ ] I **would** intervene (irrespective of the amount of displacement)

If you would intervene only with a certain amount of displacement, then what would be your threshold of displacement for intervention?

(Please answer in mm)

[ ]

7. At what measurement of displacement of **ulnar variance** would you intervene surgically?

- [ ] I **would not** intervene (irrespective of the amount of displacement)
- [ ] I **would** intervene (irrespective of the amount of displacement)

If you would intervene only with a certain amount of displacement, then what would be your threshold of displacement for intervention?

(Please answer in mm and indicate with + or - for positive or negative ulnar variance respectively)

[ ]

8. At what measurement of displacement of **radial inclination** would you intervene surgically?

- [ ] I **would not** intervene (irrespective of the amount of displacement)
- [ ] I **would** intervene (irrespective of the amount of displacement)

If you would intervene only with a certain amount of displacement, then what would be your threshold of displacement for intervention?

(Please answer in degrees)

[ ]

9. At what measurement of displacement of **dorsal tilt** would you intervene surgically?

- [ ] I **would not** intervene (irrespective of the amount of displacement)
- [ ] I **would** intervene (irrespective of the amount of displacement)

If you would intervene only with a certain amount of displacement, then what would be your threshold of displacement for intervention?

(Please answer in degrees and use negative values to indicate dorsal angulation)

[ ]

10. Please enter any comments regarding this case not captured above.

Case 2: A **58 year old** patient sustains a displaced **extra-articular** fracture of the distal radius.

* 11. ***(Please note that the age of the patient is different from that of the previous questions.)***

How important is restoring **radial height** to the position prior to injury to prevent an adverse effect on functional outcome for this patient?

Please rate on the scale (0=extremely unimportant, 10=extremely important)
Click on the line at the appropriate position, or enter a numerical rating in the textbox.

0 **Importance of Radial Height** **10**

* 12. How important is restoring **ulnar variance** to the position prior to injury to prevent an adverse effect on functional outcome for this patient?

Please rate on the scale (0=extremely unimportant, 10=extremely important)
Click on the line at the appropriate position, or enter a numerical rating in the textbox.

0 **Importance of Ulnar Variance** **10**

* 13. How important is restoring **radial inclination** to the position prior to injury to prevent an adverse effect on functional outcome for this patient?

Please rate on the scale (0=extremely unimportant, 10=extremely important)
Click on the line at the appropriate position, or enter a numerical rating in the textbox.

0	**Importance of Radial Inclination**	10	
○			☐

* 14. How important is restoring **dorsal tilt** to the position prior to injury to prevent an adverse effect on functional outcome for this patient?

Please rate on the scale (0=extremely unimportant, 10=extremely important)
Click on the line at the appropriate position, or enter a numerical rating in the textbox.

0	**Importance of Dorsal Tilt**	10	
○			☐

15. At what measurement of displacement of **radial height** would you intervene surgically?

☐ I **would not** intervene (irrespective of the amount of displacement)

☐ I **would** intervene (irrespective of the amount of displacement)

If you would intervene only with a certain amount of displacement, then what would be your threshold of displacement for intervention?

(Please answer in mm)

16. At what measurement of displacement of **ulnar variance** would you intervene surgically?

☐ I **would not** intervene (irrespective of the amount of displacement)

☐ I **would** intervene (irrespective of the amount of displacement)

If you would intervene only with a certain amount of displacement, then what would be your threshold of displacement for intervention?

(Please answer in mm and indicate with + or - for positive or negative ulnar variance respectively)

17. At what measurement of displacement of **radial inclination** would you intervene surgically?

- [ ] I **would not** intervene (irrespective of the amount of displacement)
- [ ] I **would** intervene (irrespective of the amount of displacement)

If you would intervene only with a certain amount of displacement, then what would be your threshold of displacement for intervention?

(Please answer in degrees)

18. At what measurement of displacement of **dorsal tilt** would you intervene surgically?

- [ ] I **would not** intervene (irrespective of the amount of displacement)
- [ ] I **would** intervene (irrespective of the amount of displacement)

If you would intervene only with a certain amount of displacement, then what would be your threshold of displacement for intervention?

(Please answer in degrees and use negative values to indicate dorsal angulation)

19. Please enter any comments regarding this case not captured above.

Case 3: A **75 year old** patient sustains a displaced **extra-articular** fracture of the distal radius.

* 20. ***(Please note that the age of the patient is different from that of the previous questions)***

How important is restoring **radial height** to the position prior to injury to prevent an adverse effect on functional outcome for this patient?

Please rate on the scale (0=extremely unimportant, 10=extremely important)
Click on the line at the appropriate position, or enter a numerical rating in the textbox.

0	**Importance of Radial Height**	10	

* 21. How important is restoring **ulnar variance** to the position prior to injury to prevent an adverse effect on functional outcome for this patient?

Please rate on the scale (0=extremely unimportant, 10=extremely important)
Click on the line at the appropriate position, or enter a numerical rating in the textbox.

0	**Importance of Ulnar Variance**	10	

* 22. How important is restoring **radial inclination** to the position prior to injury to prevent an adverse effect on functional outcome for this patient?

Please rate on the scale (0=extremely unimportant, 10=extremely important)
Click on the line at the appropriate position, or enter a numerical rating in the textbox.

0	**Importance of Radial Inclination**	10	

* 23. How important is restoring **dorsal tilt** to the position prior to injury to prevent an adverse effect on functional outcome for this patient?

Please rate on the scale (0=extremely unimportant, 10=extremely important)
Click on the line at the appropriate position, or enter a numerical rating in the textbox.

0	**Importance of Dorsal Tilt**	10	

24. At what measurement of displacement of **radial height** would you intervene surgically?

- [ ] I **would not** intervene (irrespective of the amount of displacement)
- [ ] I **would** intervene (irrespective of the amount of displacement)

If you would intervene only with a certain amount of displacement, then what would be your threshold of displacement for intervention?

(Please answer in mm)

25. At what measurement of displacement of **ulnar variance** would you intervene surgically?

- [ ] I **would not** intervene (irrespective of the amount of displacement)
- [ ] I **would** intervene (irrespective of the amount of displacement)

If you would intervene only with a certain amount of displacement, then what would be your threshold of displacement for intervention?

(Please answer in mm and indicate with + or - for positive or negative ulnar variance respectively)

26. At what measurement of displacement of **radial inclination** would you intervene surgically?

- [ ] I **would not** intervene (irrespective of the amount of displacement)
- [ ] I **would** intervene (irrespective of the amount of displacement)

If you would intervene only with a certain amount of displacement, then what would be your threshold of displacement for intervention?

(Please answer in degrees)

27. At what measurement of displacement of **dorsal tilt** would you intervene surgically?

- [ ] I **would not** intervene (irrespective of the amount of displacement)
- [ ] I **would** intervene (irrespective of the amount of displacement)

If you would intervene only with a certain amount of displacement, then what would be your threshold of displacement for intervention?

(Please answer in degrees and use negative values to indicate dorsal angulation)

28. Please enter any comments regarding this case not captured above.

Intra-Articular Fractures of the Distal Radius

This page asks about the measurements affecting **Intra-Articular** Fractures. There are 3 cases of patients of varied ages.

* 29. Case 4: A **38 year old patient** sustains a displaced **intra-articular fracture** of the distal radius.

(Please note that both the injury and age of the patient are now different.)

How important is reducing the **intra-articular step** to prevent an adverse effect on functional outcome for this patient?

Please rate on the scale (0=extremely unimportant, 10=extremely important)
Click on the line at the appropriate position, or enter a numerical rating in the textbox.

0	**Importance of Step**	10	

* 30. How important is reducing the **intra-articular gap** to prevent an adverse effect on functional outcome for this patient?

Please rate on the scale (0=extremely unimportant, 10=extremely important)
Click on the line at the appropriate position, or enter a numerical rating in the textbox.

0	**Importance of Gap**	10	

31. At what measurement of displacement of **intra-articular step** would you intervene surgically?

☐ I **would not** intervene (irrespective of the amount of displacement)

☐ I **would** intervene (irrespective of the amount of displacement)

If you would intervene only with a certain amount of displacement, then what would be your threshold of displacement for intervention?

(Please answer in mm)

32. At what measurement of displacement of **intra-articular gap** would you intervene surgically?

☐ I **would not** intervene (irrespective of the amount of displacement)

☐ I **would** intervene (irrespective of the amount of displacement)

If you would intervene only with a certain amount of displacement, then what would be your threshold of displacement for intervention?

(Please answer in mm)

33. Please enter any comments regarding this case not captured above.

* 34. Case 5: A **58 year old** patient sustains a displaced **intra-articular fracture** of the distal radius.

(Please note that the age of the patient is different from that of the previous questions)

How important is reducing the **intra-articular step** to prevent an adverse effect on functional outcome for this patient?

Please rate on the scale (0=extremely unimportant, 10=extremely important)
Click on the line at the appropriate position, or enter a numerical rating in the textbox.

0 **Importance of Step** **10**

* 35. How important is reducing the **intra-articular gap** to prevent an adverse effect on functional outcome for this patient?

Please rate on the scale (0=extremely unimportant, 10=extremely important)
Click on the line at the appropriate position, or enter a numerical rating in the textbox.

0 **Importance of Gap** **10**

36. At what measurement of displacement of **intra-articular step** would you intervene surgically?

- [ ] I **would not** intervene (irrespective of the amount of displacement)
- [ ] I **would** intervene (irrespective of the amount of displacement)

If you would intervene only with a certain amount of displacement, then what would be your threshold of displacement for intervention?

(Please answer in mm)

37. At what measurement of displacement of **intra-articular gap** would you intervene surgically?

- [ ] I **would not** intervene (irrespective of the amount of displacement)
- [ ] I **would** intervene (irrespective of the amount of displacement)

If you would intervene only with a certain amount of displacement, then what would be your threshold of displacement for intervention?

(Please answer in mm)

38. Please enter any comments regarding this case not captured above.

* 39. Case 6: A **75 year old** patient sustains a displaced **intra-articular fracture** of the distal radius.

(Please note that the age of the patient is different from that of the previous questions)

How important is reducing the **intra-articular step** to prevent an adverse effect on functional outcome for this patient?

Please rate on the scale (0=extremely unimportant, 10=extremely important)
Click on the line at the appropriate position, or enter a numerical rating in the textbox.

0	**Importance of Step**	10

* 40. How important is reducing the **intra-articular gap** to prevent an adverse effect on functional outcome for this patient?

Please rate on the scale (0=extremely unimportant, 10=extremely important)
Click on the line at the appropriate position, or enter a numerical rating in the textbox.

0	**Importance of Gap**	10

41. At what measurement of displacement of **intra-articular step** would you intervene surgically?

☐ I **would not** intervene (irrespective of the amount of displacement)

☐ I **would** intervene (irrespective of the amount of displacement)

If you would intervene only with a certain amount of displacement, then what would be your threshold of displacement for intervention?

(Please answer in mm)

42. At what measurement of displacement of **intra-articular gap** would you intervene surgically?

☐ I **would not** intervene (irrespective of the amount of displacement)

☐ I **would** intervene (irrespective of the amount of displacement)

If you would intervene only with a certain amount of displacement, then what would be your threshold of displacement for intervention?

(Please answer in mm)

43. Please enter any comments regarding this case not captured above.

ATOMS
Academic Team of Musculoskeletal Surgery

Defining displacement thresholds for surgical intervention for distal radius fractures - a Delphi study

Factors affecting Surgical Intervention

This final page asks how important certain factors are, compared to similar demographics.

* 44. When deciding on whether to offer surgical intervention to a patient with a displaced distal radius fracture, how important is the following factor when deciding whether to intervene?

Medical co-morbidities

Please rate on the scale (10=extremely important, 0=extremely unimportant)
Click on the line at the appropriate position, or enter a numerical rating in the textbox.

0 **Medical co-morbidities** **10**

45. Please enter any additional comments regarding this factor

* 46. When deciding on whether to offer surgical intervention to a patient with a displaced distal radius fracture, how important is the following factor when deciding whether to intervene?

Likely compliance with rehabilitation

Please rate on the scale (10=extremely important, 0=extremely unimportant)
Click on the line at the appropriate position, or enter a numerical rating in the textbox.

0 **Likely compliance with rehabilitation** **10**

47. Please enter any additional comments regarding this factor

* 48. When deciding on whether to offer surgical intervention to a patient with a displaced distal radius fracture, how important is the following factor when deciding whether to intervene?

Age

Please rate on the scale (10=extremely important, 0=extremely unimportant)
Click on the line at the appropriate position, or enter a numerical rating in the textbox.

0 **Age** **10**

49. Please enter any additional comments regarding this factor

* 50. When deciding on whether to offer surgical intervention to a patient with a displaced distal radius fracture, how important is the following factor when deciding whether to intervene?

Gender

Please rate on the scale (10=extremely important, 0=extremely unimportant)
Click on the line at the appropriate position, or enter a numerical rating in the textbox.

0	Gender	10	

51. Please enter any additional comments regarding this factor

* 52. When deciding on whether to offer surgical intervention to a patient with a displaced distal radius fracture, how important is the following factor when deciding whether to intervene?

Dementia / mental capacity affecting the understanding of the injury, treatment, and rehabilitation

Please rate on the scale (10=extremely important, 0=extremely unimportant)
Click on the line at the appropriate position, or enter a numerical rating in the textbox.

0	Dementia / mental capacity affecting the understanding of the injury, treatment, and rehabilitation	10	

53. Please enter any additional comments regarding this factor

* 54. When deciding on whether to offer surgical intervention to a patient with a displaced distal radius fracture, how important is the following factor when deciding whether to intervene?

Pre injury functional status

Please rate on the scale (10=extremely important, 0=extremely unimportant)
Click on the line at the appropriate position, or enter a numerical rating in the textbox.

0	Pre injury functional status	10

55. Please enter any additional comments regarding this factor

* 56. When deciding on whether to offer surgical intervention to a patient with a displaced distal radius fracture, how important is the following factor when deciding whether to intervene?

Occupation

Please rate on the scale (10=extremely important, 0=extremely unimportant)
Click on the line at the appropriate position, or enter a numerical rating in the textbox.

0	Occupation	10

57. Please enter any additional comments regarding this factor

* 58. When deciding on whether to offer surgical intervention to a patient with a displaced distal radius fracture, how important is the following factor when deciding whether to intervene?

Smoking status

Please rate on the scale (10=extremely important, 0=extremely unimportant)
Click on the line at the appropriate position, or enter a numerical rating in the textbox.

0	Smoking status	10

59. Please enter any additional comments regarding this factor

* 60. When deciding on whether to offer surgical intervention to a patient with a displaced distal radius fracture, how important is the following factor when deciding whether to intervene?

Alcohol intake

Please rate on the scale (10=extremely important, 0=extremely unimportant)
Click on the line at the appropriate position, or enter a numerical rating in the textbox.

0	Alcohol intake	10

61. Please enter any additional comments regarding this factor

* 62. When deciding on whether to offer surgical intervention to a patient with a displaced distal radius fracture, how important is the following factor when deciding whether to intervene?

Bone fragility (osteoporosis)

Please rate on the scale (10=extremely important, 0=extremely unimportant)
Click on the line at the appropriate position, or enter a numerical rating in the textbox.

0	Bone fragility	10

63. Please enter any additional comments regarding this factor

64. Please enter any additional important factors not captured above or comments, regarding whether to surgically intervene.

ATOMS
Academic Team of Musculoskeletal Surgery

Defining displacement thresholds for surgical intervention for distal radius fractures - a Delphi study

Many thanks for the time and effort you have put in to complete this survey

The next round will begin in approximately 4 weeks and feedback from the previous round will be provided
