# Supplementary material for: Defining displacement thresholds for surgical intervention for distal radius fractures – A Delphi study
Source: PLoS One. 2019 Jan 8;14(1):e0210462. doi: 10.1371/journal.pone.0210462 (PMC6324814; doi:10.1371/journal.pone.0210462)
Supplement: S2 Appendix — (PDF) [file pone.0210462.s002.pdf]

ATOMS
Academic Team of Musculoskeletal Surgery

# Round 2

# Defining displacement thresholds for surgical intervention for distal radius fractures

# A Delphi study

## Introduction

**Thank you** for taking part in this Delphi study and for your helpful comments and suggestions.

We had an excellent response to the first round.

***It is very important for the strength of a Delphi study that participants who completed the first round also complete the remaining rounds so we would be very grateful for your continued support.***

In this round we will present results from the first round and offer choices based on these results to help us achieve consensus.

First round results are displayed with bars which demonstrate the mean, median and interquartile range.

This round has 25 fewer questions than the previous round and should take considerably less time to complete.

Thank you for your time and support.

Mr Nick Johnson
Chief Investigator
Honorary Academic Fellow Trauma & Orthopaedics

Professor Joseph Dias
Professor of Hand & Orthopaedic Surgery
University Hospitals of Leicester

1. Please enter your name.

ATOMS
Academic Team of Musculoskeletal Surgery

# Round 2

# Defining displacement thresholds for surgical intervention for distal radius fractures

# A Delphi study

## **Extra-Articular** Fractures of the Distal Radius

This page asks about the factors affecting **Extra-Articular** Fractures. There are 3 cases of patients of varied ages.

Please assume all patients in the cases had normal wrist anatomy *before injury* with parameters of the following values:

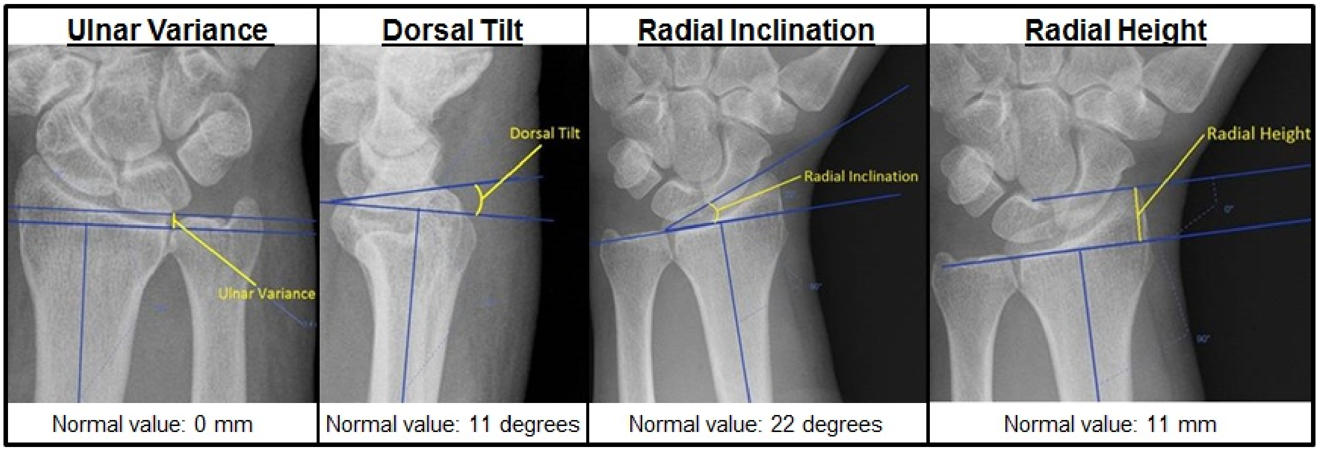


Participants were asked:

How important is restoring radial height, ulnar variance, radial inclination and dorsal tilt to the position prior to injury to prevent an adverse effect on functional outcome for this patient (0=extremely unimportant, 10=extremely important)?

Parameters were ranked in order of importance by first round mean score.

Results for each age group are shown below.

38 year old patient

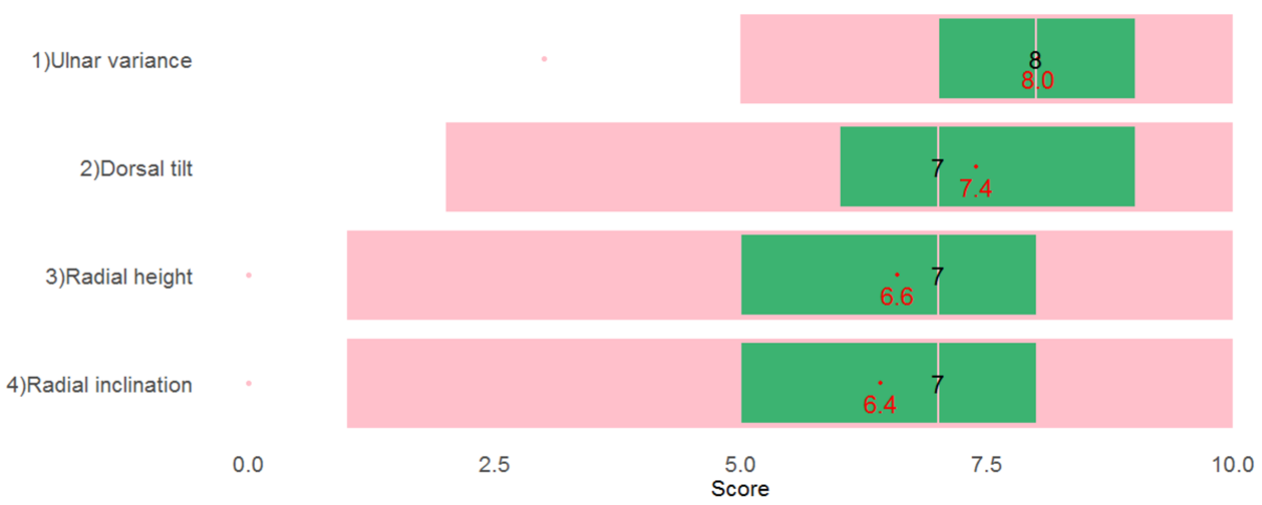


58 year old patient

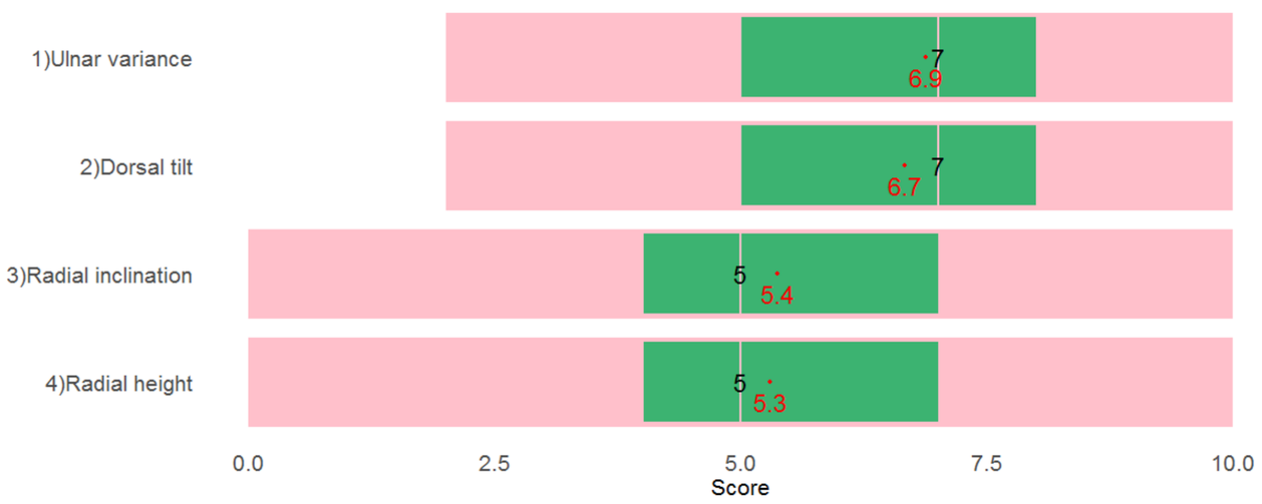


75 year old patient

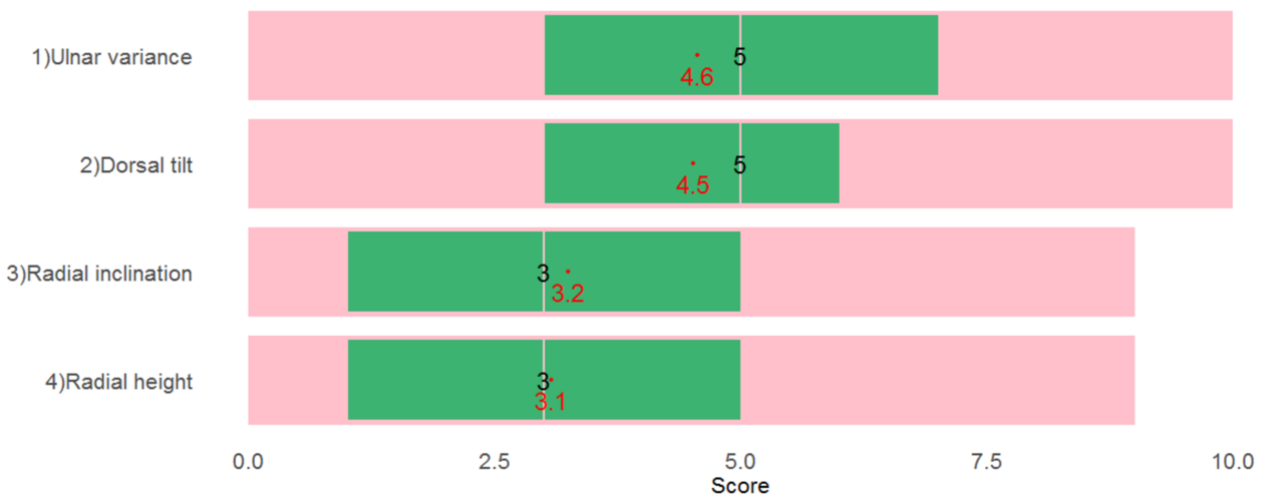

Due to the consistency and small differences between parameters for the different age groups we have combined the results of all age groups and these overall results are presented below.

**Combined results**

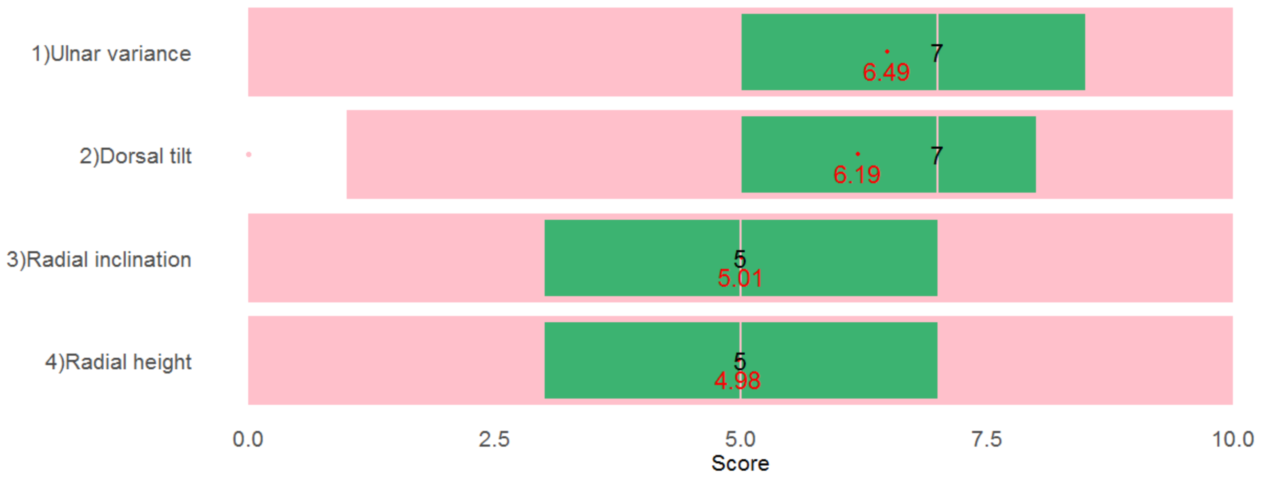


* 2. **Do you agree with this combined ranking order?**

- **Yes**
- **No**

3. Please enter any comments.

*Questions 4 and 5 are optional but we would be very interested to hear your views on these findings identified in round 1.*

4. Radial height and radial inclination were consistently considered the least important parameters by participants for each age scenario. Correlation between these factors was very high.

Do you think it is necessary to measure both of these factors?

Do you take both factors into consideration when deciding how to manage a displaced distal radius fracture?

If you use only one which one do you prefer to look at?

5. In the comments section of Round 1 several participants pointed out that carpal alignment was another important factor when deciding whether to correct dorsal tilt.

Does carpal alignment influence your decision to intervene?

If it does can you explain how it changes your decision?

ATOMS
Academic Team of Musculoskeletal Surgery

**Round 2**

**Defining displacement thresholds for surgical intervention for distal radius fractures**

**A Delphi study**

6. Please **rank in order of importance** of restoring the following 4 parameters to the position prior to injury to prevent an adverse effect on functional outcome for this patient

- **Ulnar variance**
- **Dorsal tilt**
- **Radial inclination**
- **Radial height**

The following questions are designed to move towards consensus regarding **absolute values** at which surgeons feel intervention is required.

The answer options provided were rounded to common values which would be measurable and used in clinical practice.

## Case 1: A **38 year old** patient sustains a displaced **extra-articular** fracture of the distal radius.

**Ulnar variance**

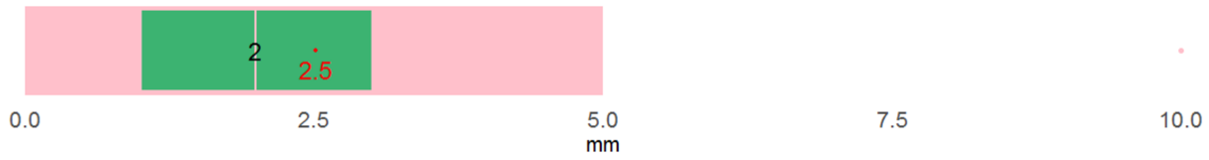

* 7. At what measurement of **ulnar variance** would you intervene surgically?

◯ **<1mm** ◯ **1mm** ◯ **2mm** ◯ **3mm** ◯ **>3mm**

## Dorsal tilt

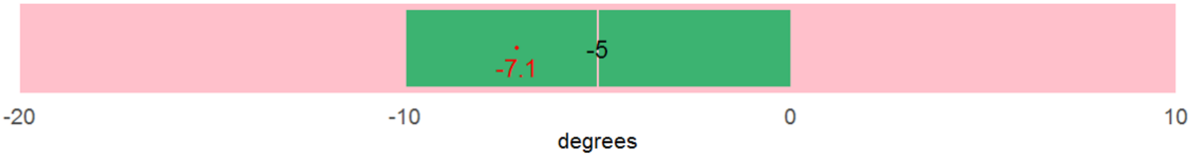


* 8. At what measurement of **dorsal tilt** would you intervene surgically?

***(negative values indicate dorsal angulation)***

◯ **>0 degrees** ◯ **0 degrees** ◯ **- 5 degrees** ◯ **-10 degrees** ◯ **<-10 degrees**

## Radial inclination

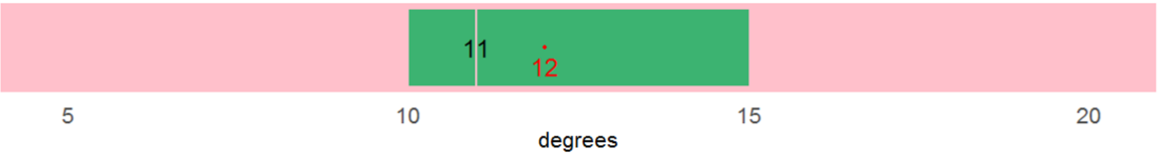


* 9. At what measurement of **radial inclination** would you intervene surgically

◯ **<5 degrees** ◯ **5 degrees** ◯ **10 degrees** ◯ **15 degrees** ◯ **>15 degrees**

## Radial height

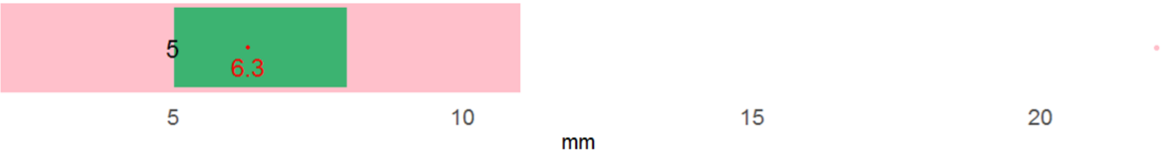


* 10. At what measurement of **radial height** would you intervene surgically?

◯ **<3mm** ◯ **3mm** ◯ **5mm** ◯ **8mm** ◯ **>8mm**

11. Please enter any comments regarding this case not captured above.

ATOMS
Academic Team of Musculoskeletal Surgery

## Round 2

## Defining displacement thresholds for surgical intervention for distal radius fractures

## A Delphi study

Case 2: A **58 year old** patient sustains a displaced **extra-articular** fracture of the distal radius.

### Ulnar variance

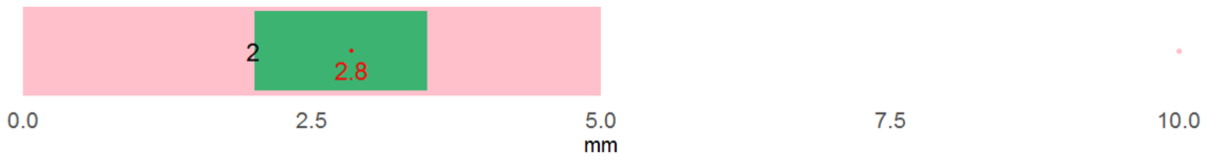


* 12. At what measurement of **ulnar variance** would you intervene surgically?

◯ **<2mm** ◯ **2mm** ◯ **3mm** ◯ **4mm** ◯ **>4mm**

### Dorsal tilt

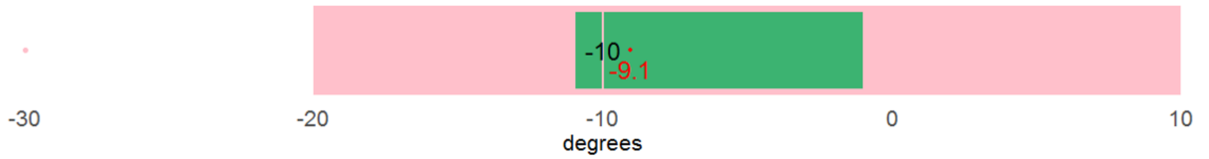


* 13. At what measurement of **dorsal tilt** would you intervene surgically?

***(negative values indicate dorsal angulation)***

◯ **<-15 degrees** ◯ **-15 degrees** ◯ **-10 degrees** ◯ **5 degrees** ◯ **>5 degrees**

**Radial inclination**

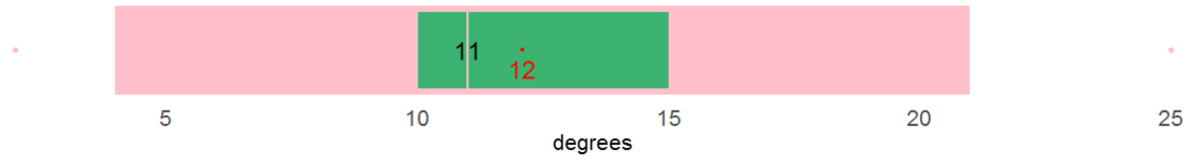


* 14. At what measurement of **radial inclination** would you intervene surgically?

◯ **<5 degrees** ◯ **5 degrees** ◯ **10 degrees** ◯ **15 degrees** ◯ **>15 degrees**

**Radial height**

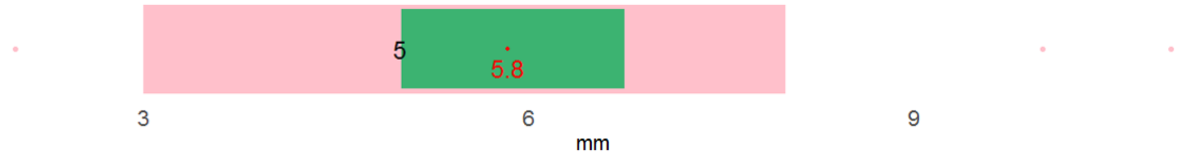


* 15. At what measurement of **radial height** would you intervene surgically?

◯ **<3mm** ◯ **3mm** ◯ **5mm** ◯ **8mm** ◯ **>8mm**

16. Please enter any comments regarding this case not captured above.

Case 3: A **75 year old** patient sustains a displaced **extra-articular** fracture of the distal radius.

**Ulnar variance**

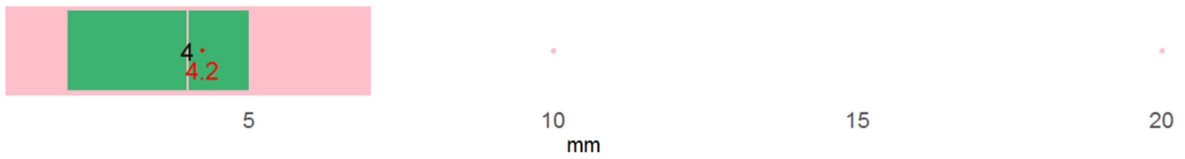


* 17. At what measurement of **ulnar variance** would you intervene surgically?

- ◯ **<2mm** ◯ **2mm** ◯ **4mm** ◯ **5mm** ◯ **>5mm**

**Dorsal tilt**

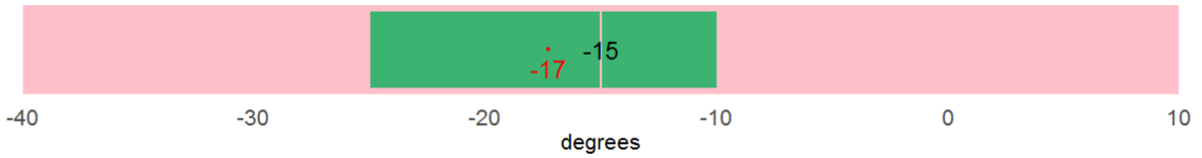


* 18. At what measurement of **dorsal tilt** would you intervene surgically?
***(negative values indicate dorsal angulation)***

- ◯ **<-30 degrees** ◯ **-30 degrees** ◯ **-20 degrees** ◯ **-10 degrees** ◯ **>0 degrees**

**Radial inclination**

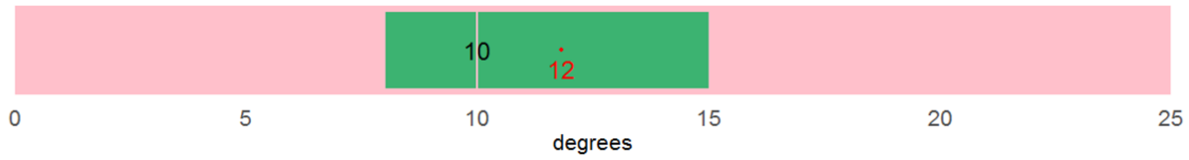


* 19. At what measurement of **radial inclination** would you intervene surgically?

◯ **<5 degrees** ◯ **5 degrees** ◯ **10 degrees** ◯ **15 degrees** ◯ **>15 degrees**

**Radial height**

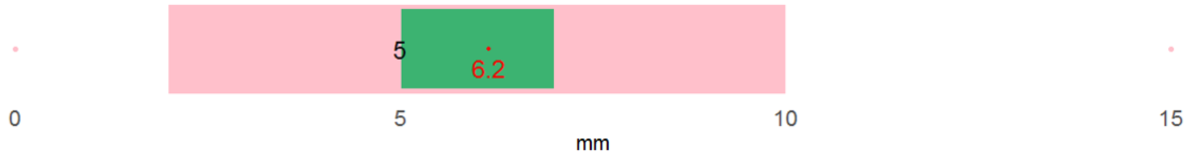


* 20. At what measurement of **radial height** would you intervene surgically?

◯ **<5mm** ◯ **5mm** ◯ **10mm** ◯ **15mm** ◯ **>15mm**

21. Please enter any comments regarding this case not captured above.

Round 2

# Defining displacement thresholds for surgical intervention for distal radius fractures

A Delphi study

## **Intra-Articular** Fractures of the Distal Radius

This page asks about the measurements affecting **Intra-Articular** Fractures. There are 3 cases of patients of varied ages.

Participants were asked:
How important is restoring intra-articular step and gap to the position prior to injury to prevent an adverse effect on functional outcome for this patient (0=extremely unimportant, 10=extremely important)?

Parameters were ranked in order of importance by first round mean score.
Results for each age group are shown below.

**38 year old patient**

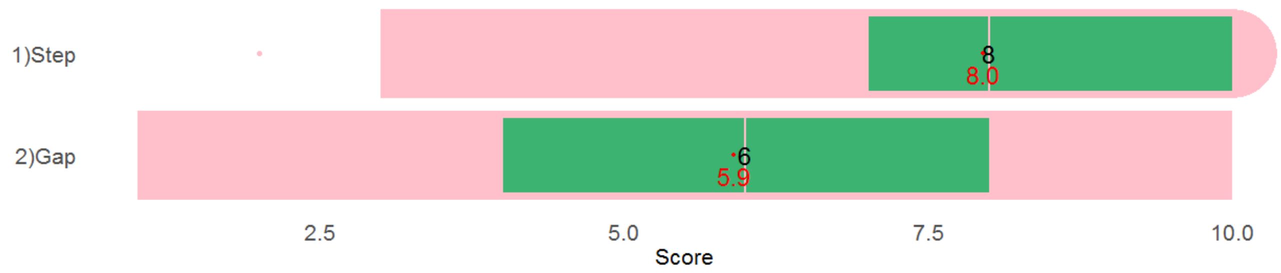


**58 year old patient**

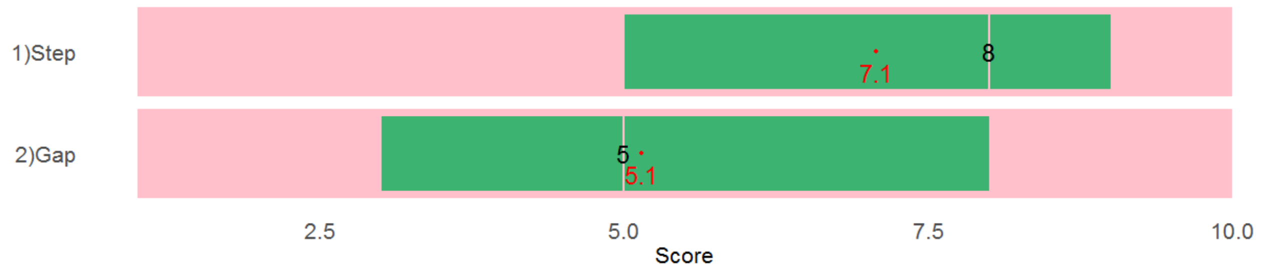

**75 year old patient**

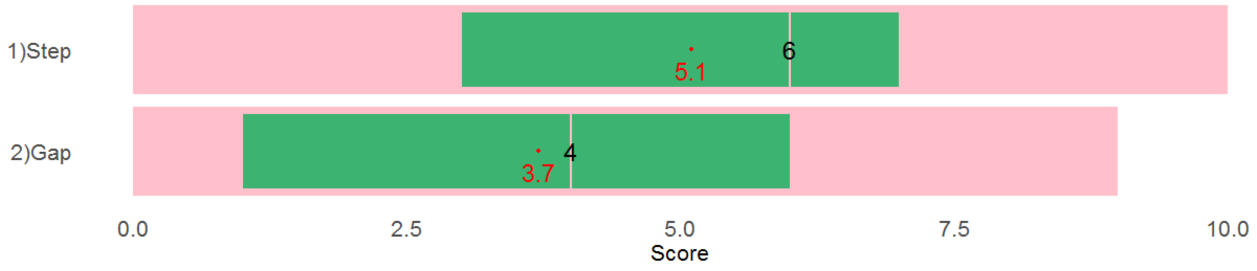


Due to the consistency of ranking order between parameters for the different age groups we have combined the results of all age groups and these overall results are presented below.

**Combined results**

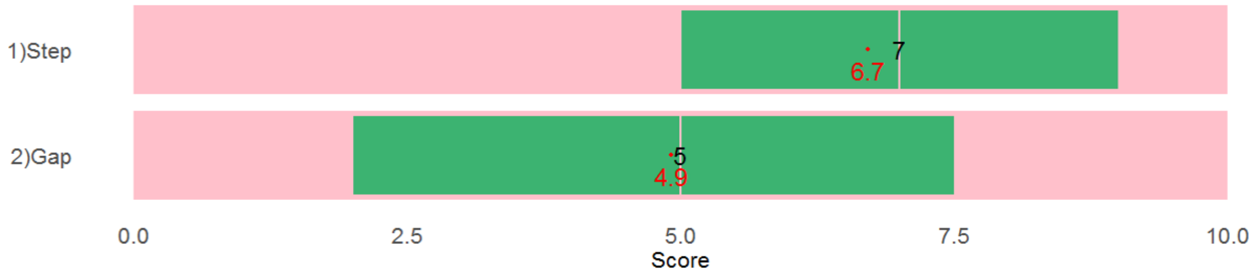


* 22. **Do you agree with this combined ranking order?**

◯ **Yes**

◯ **No**

23. Please enter any comments.

The following questions are designed to move towards consensus regarding absolute values at which surgeons feel intervention is required.

Case 4: A **38 year old** patient sustains a displaced **intra-articular** fracture of the distal radius.

**Intra-articular step**

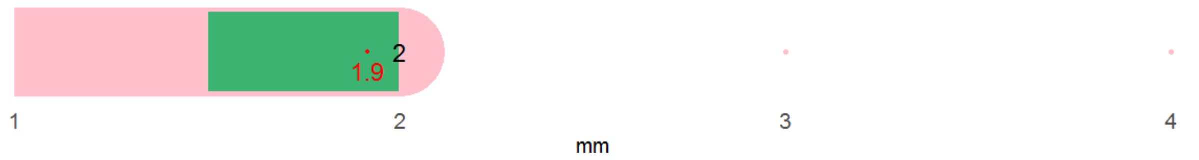


* 24. At what measurement of **intra-articular step** would you intervene surgically?

◯ **<1mm** ◯ **1mm** ◯ **2mm** ◯ **3mm** ◯ **>3mm**

**Intra-articular gap**

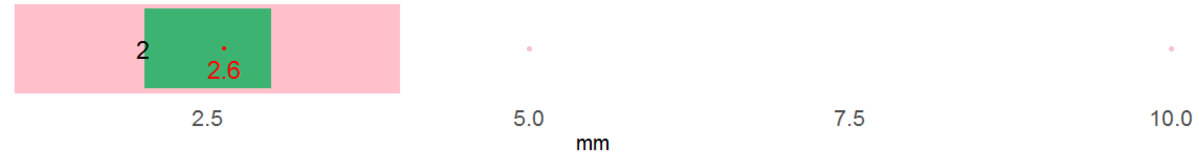


* 25. At what measurement of **intra-articular gap** would you intervene surgically?

◯ **<2mm** ◯ **2mm** ◯ **3mm** ◯ **4mm** ◯ **>4mm**

26. Please enter any comments regarding this case not captured above.

Case 5: A **58 year old** patient sustains a displaced **intra-articular** fracture of the distal radius.

**Intra-articular step**

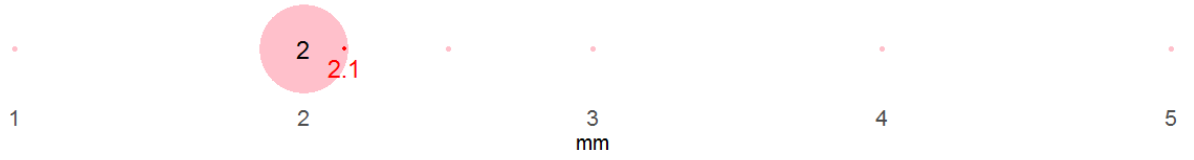


* 27. At what measurement of **intra-articular step** would you intervene surgically?

○ **<1mm** ○ **1mm** ○ **2mm** ○ **3mm** ○ **>3mm**

**Intra-articular gap**

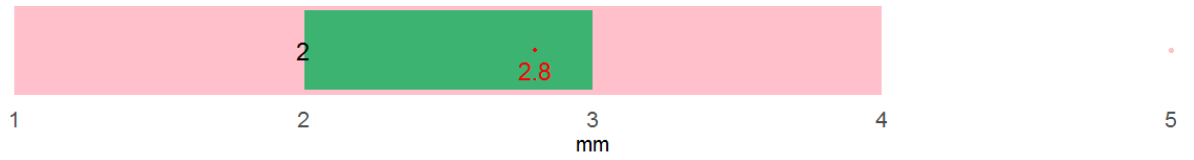


* 28. At what measurement of **intra-articular gap** would you intervene surgically?

○ **<2mm** ○ **2mm** ○ **3mm** ○ **4mm** ○ **>4mm**

29. Please enter any comments regarding this case not captured above.

[ ]

Round 2

Defining displacement thresholds for surgical intervention for distal radius fractures

A Delphi study

Case 6: A **75 year old** patient sustains a displaced **intra-articular** fracture of the distal radius.

**Intra-articular step**

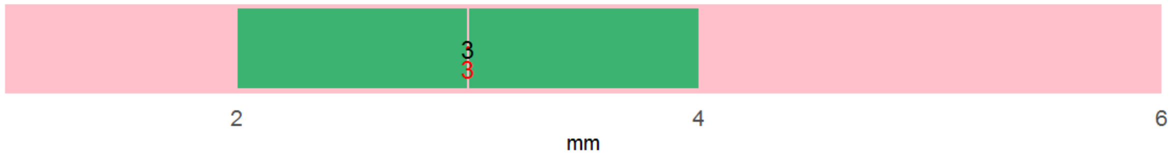


* 30. At what measurement of **intra-articular step** would you intervene surgically?

◯ **<2mm** ◯ **2mm** ◯ **3mm** ◯ **4mm** ◯ **>4mm**

**Intra-articular gap**

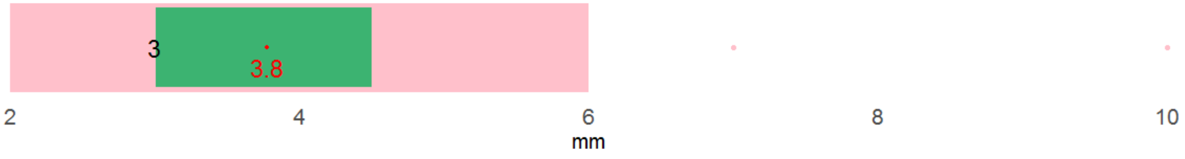


* 31. At what measurement of **intra-articular gap** would you intervene surgically?

◯ **<3mm** ◯ **3mm** ◯ **4mm** ◯ **5mm** ◯ **>5mm**

32. Please enter any comments regarding this case not captured above.

ATOMS
Academic Team of Musculoskeletal Surgery

# Round 2

# Defining displacement thresholds for surgical intervention for distal radius fractures

# A Delphi study

## Factors affecting Surgical Intervention

This question investigates the importance of patient factors when deciding whether to intervene.

In the first round consensus was obtained that **dementia / mental capacity, pre-injury function and medical co-morbidities** were the most important factors.

**Gender, smoking and alcohol intake** were not important.

The results from the previous round are shown below in order of importance.

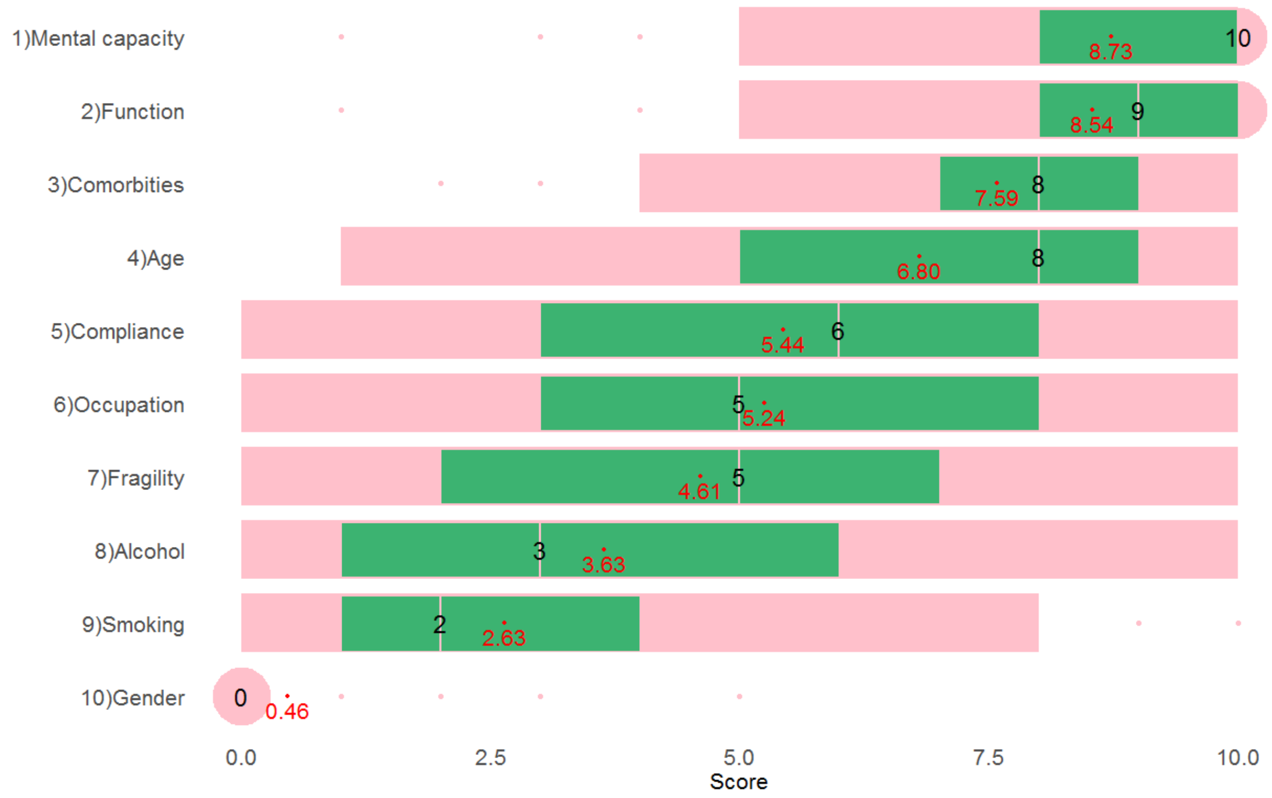

33. If you have any comments regarding these results please add them here.

To help obtain consensus on the importance on the remaining four factors please re-score them.

*We would encourage you to be bold with the scoring*.

* 34. When deciding on whether to offer surgical intervention to a patient with a displaced distal radius fracture, how important is the following factor when deciding whether to intervene?
**Age**
Please rate on the scale (0=extremely unimportant, 10=extremely important)
Click on the line at the appropriate position, or enter a numerical rating in the textbox.

**0** **Age** **10**

* 35. When deciding on whether to offer surgical intervention to a patient with a displaced distal radius fracture, how important is the following factor when deciding whether to intervene?
**Likely compliance with rehabilitation**
Please rate on the scale (0=extremely unimportant, 10=extremely important)
Click on the line at the appropriate position, or enter a numerical rating in the textbox.

**0** **Likely compliance with rehabilitation** **10**

* 36. When deciding on whether to offer surgical intervention to a patient with a displaced distal radius fracture, how important is the following factor when deciding whether to intervene?
**Occupation**
Please rate on the scale (0=extremely unimportant, 10=extremely important)
Click on the line at the appropriate position, or enter a numerical rating in the textbox.

**0** **Occupation** **10**

* 37. When deciding on whether to offer surgical intervention to a patient with a displaced distal radius fracture, how important is the following factor when deciding whether to intervene?

**Bone fragility (osteoporosis)**

Please rate on the scale (0=extremely unimportant, 10=extremely important)

Click on the line at the appropriate position, or enter a numerical rating in the textbox.

**0** **Bone fragility** **10**

38. Please enter any additional comments here.

ATOMS
Academic Team of Musculoskeletal Surgery

## Round 2

## Defining displacement thresholds for surgical intervention for distal radius fractures

## A Delphi study

Many thanks for the time and effort you have put in to complete this survey

The third and final round will begin in approximately 4 weeks and feedback from the previous round will be provided
