# Supplementary material for: Defining displacement thresholds for surgical intervention for distal radius fractures – A Delphi study
Source: PLoS One. 2019 Jan 8;14(1):e0210462. doi: 10.1371/journal.pone.0210462 (PMC6324814; doi:10.1371/journal.pone.0210462)
Supplement: S3 Appendix — (PDF) [file pone.0210462.s003.pdf]

ATOMS
Academic Team of Musculoskeletal Surgery

Round 3

Defining displacement thresholds for surgical intervention for distal radius fractures

A Delphi study

Introduction

Thank you for taking part in this Delphi study and for your continued support.

Through this iterative Delphi process we have explored the expert panel's views on:

1) The rank order of radiographic parameter importance
2) The relative importance of radiographic parameters
3) Radiographic thresholds for intervention
4) Factors which influence the decision to intervene

In this third and final round we wish to explore further the factors that influence decision making and how these may adjust your thresholds for intervention.

In clinical practice the treating surgeon views radiographs of the injury first and makes an initial judgement about whether displacement is likely to impair function. Additional information about the individual patient is then used to make the final decision regarding the most appropriate treatment for that patient.

The 12 questions in this round aim to help understand how a surgeon uses this patient information to inform their decision making and how

this may alter their usual radiographic thresholds for intervention.

We would be extremely grateful for your comments in the boxes provided.

Further information and feedback is provided in the additional pdfs sent to you.

We have used a traffic light system as demonstrated below to illustrate agreement and importance

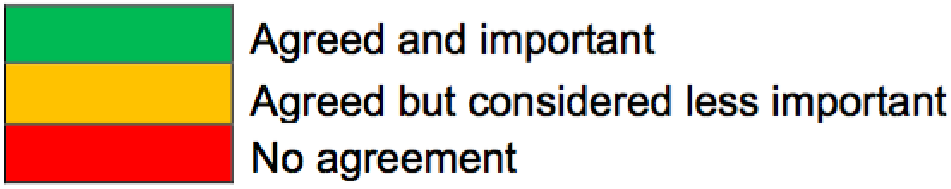

Thank you for your time and support.

Mr Nick Johnson
Chief Investigator
Honorary Academic Fellow Trauma & Orthopaedics

Professor Joseph Dias
Professor of Hand & Orthopaedic Surgery
University Hospitals of Leicester

1. Please enter your name.

2. For how many years have you been practising as a consultant/attending surgeon?

ATOMS
Academic Team of Musculoskeletal Surgery

Round 3

Defining displacement thresholds for surgical intervention for distal radius fractures

A Delphi study

Ranking of importance of radiographic parameters

	Parameter	
Ranking	Extra-articular	Intra-articular
1	Ulnar variance	Step
2	Dorsal tilt	Gap
3	Radial inclination	
4	Radial height	

Thresholds for intervention

Agreement denotes the percentage of the expert panel who would intervene at this radiographic threshold

Patient age	Extra-articular parameters: Ulnar variance (mm)	Dorsal tilt (degrees)	Radial inclination (degrees)	Radial height (degrees)
38	3	10	10	5
agreement	84%	79%	90%	85%
58	3	10	10	5
agreement	74%	87%	82%	90%
75	4 / >5	20	10	5
agreement	50% / 42%	87%	91%	88%

Patient age	Intra-articular parameters: Step (mm)	Gap (mm)
38	2	3
agreement	81%	84%
58	2	3
agreement	76%	87%
75	3	4
agreement	76%	79%

The panel agreed they would intervene at the same threshold for patients aged 38 and 58 years for each radiographic parameter.

3. Please enter any comments

ATOMS
Academic Team of Musculoskeletal Surgery

Round 3

Defining displacement thresholds for surgical intervention for distal radius fractures

A Delphi study

<u>Ulnar variance in a 75 year old patient</u>

Agreement was not reached for this question.

· 50% of participants would intervene at 4mm of positive ulnar variance

· 42% would accept 5mm or greater of positive ulnar variance

Although literature suggests shortening in the form of positive ulnar variance is associated with a poorer outcome the effect is less certain in older patients.

Patients ≥65 years of age showed no statistically significant relationship between malalignment of the distal radius and PRWE or DASH scores when radiography parameters were examined in isolation and when clustered together. 216 patients with an extra-articular distal radius fracture were followed up for one year.
Grewal R et al;The risk of adverse outcomes in extra-articular distal radius fractures is increased with malalignment in patients of all ages but mitigated in older patients. J Hand Surg [Am], 2007.

Ulnar variance and volar tilt are the most important radiographic parameters to be restored to obtain good functional outcome in distal radius fracture. Small variations of other radiographic parameters seem to not affect the final outcome at minimum 3 years' follow-up in 51 patients treated with volar locking plates.
Dario P et al; Is it really necessary to restore radial anatomic parameters after distal radius fractures? Injury, 2014.

Positive ulnar variance of > 3 mm resulted in reduced grip strength in a prospective randomised trial of 120 patients of mean age 63 years (range 16 to 86) with re-displaced distal radius fractures.
McQueen MM et al; Redisplaced unstable fractures of the distal radius: a prospective randomised comparison of four methods of treatment. J Bone Joint Surg [Br], 1996

4. Taking the panel results and literature into consideration:

Please give us your views regarding ulnar variance in a ***fit, compliant*** 75 year old?

Do we need a threshold for ulnar variance in this age group?

Any other comments?

ATOMS
Academic Team of Musculoskeletal Surgery

Round 3

Defining displacement thresholds for surgical intervention for distal radius fractures

A Delphi study

Carpal malalignment/displacement was identified as an additional important factor by 74% of participants.

Carpal malalignment is defined as the displacement of the longitudinal axis of the capitate either dorsal or volar to the longitudinal axis of the radius.

Taleisnik J et al; Midcarpal instability caused by malunited fractures of the distal radius. J Hand Surg Am, 1984

101 patients with a unilateral distal radius fracture had radiographs taken of both wrists 3 months after injury. Change in the orientation of the distal articular surface of the radius adversely influenced the intercarpal relationship with loss of volar tilt causing the lunate to dorsiflex and the capitate go into a compensatory flexion pattern.

Dias JJ et al; Effect of Colles fracture malunion on carpal alignment. R Col Surg Edin, 1988.

Carpal malalignment was an indicator of poor function using a modified scoring system incorporating pain, range of movement and grip strength in a case series of 69 patients studied at 1 year after injury investigating factors that may affect functional outcome.

Batra S et al; The effect of fracture-related factors on the functional outcome at 1 year in distal radius fractures. Injury, 2002.

Carpal malalignment was the major influence on function and associated with diminished recovery of grip strength and range of movement. It was seen in 50 (42%) out of 120 wrists after union. Of these 31 (62%) had malunited in dorsal tilt (>10 degrees).

McQueen MM et al; Redisplaced unstable fractures of the distal radius: a prospective randomised comparison of four methods of treatment. J Bone Joint Surg [Br], 1996.

Carpal malalignment reflects loss of volar tilt. Several studies have shown it is associated with poorer functional outcome.

5. How would you manage a patient with carpal malalignment and an acute distal radius fracture with dorsal tilt of less than 10 degrees?

ATOMS
Academic Team of Musculoskeletal Surgery

Round 3

Defining displacement thresholds for surgical intervention for distal radius fractures

A Delphi study

Decision influencing patient factors

Ranking of importance of factors

7 parameters were identified as important by the panel

Consensus was gained on the following ranking order

1)mental capacity
2)function
3)medical comorbidities
4)age
5)compliance with rehabilitation
6)occupation
7)fragility

In the following questions we want to explore how you use these factors to modify your treatment thresholds.

Please fill out as many of the following questions as possible, particularly about factors you use in your decision making.

6. Mental capacity

We would expect dementia or reduced mental capacity would increase a surgeon's radiographic thresholds for intervention.

How would you establish mental capacity in a patient with a distal radius fracture?

Why do you think mental capacity is ranked as the most important factor?

7. Function

We would expect poor pre-injury function would increase a surgeon's radiographic thresholds for intervention.

How do you define function in a patient with a distal radius fracture?

Why do you think function is ranked as the second most important factor?

8. Comorbidity

We would expect when treating a patient with multiple medical comorbidities a surgeon's radiographic thresholds for intervention would increase.

In a patient with a displaced wrist fracture and multiple co-morbidities is it problems related to anaesthetic, surgical complications or the patient's functional level that most influence your decision on whether and how to intervene?

9. Age

The results from the panel so far suggest different radiographic thresholds for intervention. Thresholds were the same for a 38 year old and 58 year old but increased for a 75 year old

Many participants mentioned the importance of 'physiological' age rather than chronological age when deciding whether to intervene.

How do you define this and how do you establish it when assessing a patient with a distal radius fracture?

10. Compliance with rehabilitation

We would expect when treating a patient with poor compliance a surgeon's radiographic thresholds for intervention would increase.

What problems regarding a patient's compliance would you be concerned about which may influence your decision to intervene and would this alter the type of intervention you would recommend?

Why do you think participants ranked this as an important factor?

11. Occupation

How would this factor influence the radiographic thresholds for intervention?

What aspects of a patients occupation influence your decision to intervene?

12. Osteoporosis/Fragility

How would this factor influence the radiographic thresholds for intervention?

How does osteoporosis/fragility influence how you would intervene?

Many thanks for the time and effort you have put in to complete this survey

We will provide further feedback in approximately 2 weeks
